# Successful Pharmacologic Treatment of Self-Bloodletting with Factitious Chronic Anemia (Lasthénie de Ferjol Syndrome) with High-Dose Serotonergic Medication: A Case Report

**DOI:** 10.3390/bs14030237

**Published:** 2024-03-14

**Authors:** Stefan Mestermann, Laura Rudtke, Razvan-Marius Brazdis, Thanos Tsaktanis, Johannes Kornhuber, Norbert Thürauf

**Affiliations:** 1 Department of Psychiatry and Psychotherapy, University Hospital Erlangen, Friedrich-Alexander-Universität Erlangen-Nürnberg (FAU), 91054 Erlangen, Germanynorbert.thuerauf@uk-erlangen.de (N.T.); 2 Department of Neurology, University Hospital Erlangen, Friedrich-Alexander-Universität Erlangen-Nürnberg (FAU), 91054 Erlangen, Germany

**Keywords:** self-injury behavior, self-harm, self-bloodletting, Lasthénie de Ferjol syndrome, mental health, clomipramine, escitalopram

## Abstract

Self-induced bloodletting (SBL) is a very rare form of self-injury (SI) seen primarily in adolescents and young adults with personality and eating disorders. It can result in complications like malaise, fatigue, or iron-deficiency anemia (Lasthénie de Ferjol syndrome, LFS), and poses a risk of accidental death or suicide. The condition often goes undetected due to patient concealment. There is no specific treatment established, and pharmacological strategies remain uncertain. We discuss the case of a 22-year-old female patient treated at our Psychiatry and Psychotherapy Department following a suicide attempt via SBL. She self-administered a venous cannula, losing 1.5 L of blood. Diagnosed with iron-deficiency anemia (LFS), she was initially treated with mirtazapine, risperidone, lithium, and later off-label high-dose clomipramine (300 mg/d). Clomipramine significantly reduced her SBL and suicidal thoughts, and her hemoglobin levels re-normalized under iron-substitution therapy. Despite improvement and later discharge, she attempted suicide by SBL again three months later, having stopped clomipramine due to adverse side effects. High-dose escitalopram was administered, leading to a decrease and eventual cessation of her SBL urges. This case demonstrates that patients with SBL/LFS can benefit from high-dose clomipramine or escitalopram. Despite its rarity, the consideration of high-dose serotonergic antidepressants is crucial in psychiatric diagnostics and treatment for patients affected by SBL/LFS.

## 1. Background

Deliberate self-injury (SI) appears as a symptom of various psychiatric disorders. The global prevalence of self-injurious behavior is around 19% among adolescents and young adults, peaking in European countries and more frequently affecting females [1]. Self-induced bloodletting (‘Self-bloodletting’ or SBL) represents a rare expression of self-harm with possible suicidal intentions, wherein patients drain their own blood via venipuncture or other forms of vessel injuries [2]. Self-injury, in general, may have different and mixed psychopathological backgrounds. Most commonly, patients report intrapersonal motives, like the wish to regulate internal tension/other stressful states or the urge for self-punishment. Interpersonal reasons, e.g., the wish to express/communicate distress to others or punish others, are less frequent [3].

Contrary to the majority of other self-harm variations, even for primarily non-suicidal SBL, there is a high risk of losing consciousness as a consequence of blood loss. Unintentional serious health impairments, such as hypoxic brain damage but also accidental death, are possible and presumably accepted by the patients as an expression of passive suicidality. The majority of patients with SBL are female, and a connection with an educational/professional background in healthcare (e.g., paramedics, nursing staff, physicians) is described [4]. Presumably, the low-level access to medical material like blood collection instruments, knowledge of venipuncture procedures, and a hypothetical fascination with the theme of blood may pose risk factors. Further somatic complications of SBL are malaise or chronic fatigue. When SBL induces factitious chronic iron-deficiency anemia, the clinical case is described as Lasthénie de Ferjol syndrome (LFS) [5].

Yet, there are only a few cases in the literature reporting SBL; data on diagnostics and treatment are lacking in psychiatric research. The psychopathology of SBL seems to be frequently undetected, as patients tend to conceal SBL. Idiopathic chronic, typically microcytic hypochromic anemia may be a clinical sign but is often not primarily linked to SBL, LFS, or self-injury in general in the clinical context [6]. 

There is no established form of pharmacological or psychosocial therapeutic strategy for SBL. German and international medical guidelines primarily recommend psychotherapeutic approaches for the treatment of SI. Recommendations for the administration of antipsychotics, selective serotonin reuptake inhibitors (SSRIs), or naltrexone remain uncertain [7,8]. For SBL, specific individual pharmacologic treatment approaches include atypical antipsychotics (AAPs), which only show weak evidence, or serotonergic agents [6], although positive effects on long-term outcomes have not been researched yet. Despite SBL being a rare psychopathologic symptom, clinicians in psychiatric healthcare would benefit from standardized pharmacological strategies for the treatment of SBL.

The objective of this article is to present the clinical case of a 22-year-old adolescent woman with repeated SBL with non-suicidal and suicidal intentions. We intend to outline the difficulties in finding effective psychiatric therapy strategies and to propose possible pharmacologic treatment options.

## 2. Case Presentation

### 2.1. Clinical Presentation and Diagnostics

A 22-year-old female patient was brought in for diagnostics and therapy on an emergency basis at our Department of Psychiatry and Psychotherapy, University Hospital of Erlangen, Germany. The day before, she had attempted suicide via SBL. The young woman, who was working as a paramedic, had self-applied a venous canula to her antecubital vein, followed by the deliberate loss of 1.5 L of her own blood while being (intentionally) intoxicated by lorazepam and LSD. She lost consciousness and was found unconscious several hours later and presented at our psychiatric emergency room. The patient presented physical weakness and fatigue. Pre-existing psychiatric disorders were recurrent depression and an eating disorder, which were first diagnosed during adolescence. Therefore, she had previously undergone child and adolescent psychiatric treatments. At inpatient admission, the current psychiatric medication was mid-dose escitalopram at 15-0-0 mg/d, which had been prescribed during earlier child and adolescent psychiatric therapy for the treatment of our patient’s depressive disorder. Later, our patient revealed a history of anamnestic self-injury and one additional previous suicide attempt, also by SBL. She specifically described experiencing a kind of emotional upswing (‘kick’) following SBL and perceived thoughts of passing out as exciting/thrilling as well as calming. Due to acute suicidality, the young woman was admitted to our psychiatric acute care unit.

The patient self-reported a low and dysphoric mood, avolition, loss of interest, anhedonia, and worrying about her own future. As no psychotic or delusional symptoms were present, the criteria for Recurrent Depressive Disorder, with a severe current episode without psychotic symptoms (ICD-10: F33.2), were met. During diagnostics and treatment, anamnesis, interaction analysis, and psychological testing revealed the diagnosis of Emotionally Unstable Personality Disorder: Borderline-type (BPD, ICD-10: F60.31). Although the acute case did not demand intravenous iron substitution or erythrocyte concentrations, blood analysis indicated iron-deficiency anemia as a consequence of repeated self-induced blood loss, meeting the criteria for LFS. Further clinical internistic and neurological diagnostics showed no somatic impairments/diseases.

### 2.2. Therapeutic Process

As intervention, the patient received a highly structured multimodal psychiatric therapy concept, including psychotherapy, ergo-, and exercise-therapy. We began additional medication with mirtazapine (0-0-0-15 mg/d), risperidone (0-0-1-0 mg/d), and later lithium–augmentation (450-0-225-0 mg/d under therapeutic drug monitoring [TDM]). Sleep disorders were addressed with daridorexant medication (0-0-0-50 mg/d). Medication was well-tolerated; muscular fasciculations and pain, particularly facial, were interpreted as adverse side effects of lithium therapy and vanished after adequate adjustment of lithium plasma concentration to 0.8 mmol/L. Yet, during inpatient treatment, our patient committed multiple self-injuries and one suicide attempt by SBL with needles and peripheral venous catheters. Thus, over months, iterated permanent personal (nursing team, the patient’s parents) or video monitoring and the application of a defined anti-SBL therapy contract were necessary. Further, in states of high tension and self-harm ideations, our patient received on-demand medication (melperone, lorazepam) at certain points. After reduction and discontinuation of eescitalopram, mirtazapin, and rispierdone and with regard to the obsessive/compulsive characteristics of our patient’s SBL, we began off-label high dose clomipramine under TDM. A steady state was accomplished with a daily dosage of 300 mg/d (Table 1). Under clomipramine, the patient’s thoughts of SBL and suicide reduced significantly. There were no more self-injuries or concealments of blood collection instruments during the remaining inpatient treatment. The hemoglobin level re-normalized under oral iron-substitution therapy, and iron-deficiency anemia subsided. We highly recommended adjustments to her work situation and advised against contact with occupational healthcare activities and instruments related to blood sampling. After psychiatric stabilization, the patient was discharged after four months of inpatient therapy. 

Three months later, she presented to us again following another suicide attempt via SBL in her domestic setting. Concrete psychosocial stress factors were not reported. Instead, our patient described a recurring and increasing ‘internal urge’ to conduct SBL, additionally triggered by renewed contact with her healthcare working environment and thus exposure to blood sampling diagnostics and instruments. Further, she had terminated clomipramine due to adverse side effects (e.g., shivering, nausea) and subjectively perceived reduction in effectiveness upon possible intake discontinuation/reduced medication adherence. The other prescribed psychiatric drugs had been continued. During subsequent inpatient treatment, additional self-harm via skin packing/cutting and once by swallowing metallic staples occurred. Punctual discontinuation of clomipramine during the outpatient treatment stage could not be excluded; yet, our patient refused re-dosing of clomipramine. Thus, we re-applied escitalopram with on-label high dosage (20-0-0 mg/d), under which compulsive thoughts of SBL and self-injury significantly decreased and then vanished. Following this, dialectical behavioral therapy was recommended, and further outpatient treatment at our department was scheduled.

## 3. Discussion and Conclusions

As there are very few clinical case reports of patients affected by SBL, little is known about adequate diagnostics and, in particular, therapeutic strategies. More common forms of self-injury are skin-cutting/picking, skin burning, or swallowing sharp objects, which our patient presented during later stages of inpatient treatment. There is only little literature about SBL and LFS, which complicates scientific contextualization. 

As there are no specific recommendations regarding the psychopharmacologic treatment of SBL and LFS, our patient’s recurring and therapy-resistant SBL behavior posed a high challenge. Although self-injury might be a symptom of depression [9], our initial anti-depressive agents with additional augmentation in accordance with S3-guidelines did not improve our patient’s symptoms. As BPD is a frequent underlying psychiatric disorder of SI [10], disorder-specific treatment approaches, such as dialectical behavioral therapy (DBT) including stress tolerance skills to reduce SI, have been proven to successfully reduce SI [11]. Yet, BPD guidelines do not include general or SBL-specific profound pharmacologic treatment approaches. Our patient suffers from comorbid BPD, yet SBL is postulated to be a specific psychopathological entity that seems to be highly therapy-resistant. It may appear plausible that atypical antipsychotics can have positive effects on self-injury [12], yet, the literature reports no evidence for a specific improvement in SBL. Also, in our case, risperidone showed no significant effect regarding SBL. Further, the risk for tardive dyskinesia is present, which needs to be considered, particularly for adolescent/young adult patients [13]. The long-term tolerability of antipsychotics in young patients is heterogenous [14]. Our patient received multiple psychopharmacologic agents; thus, it is difficult to determine which of the prescribed drugs made the difference regarding SBL. 

We decided to use an experimental strategy of high-dose off-label clomipramine, which some guidelines (e.g., the German S3-guildeline for OCD treatment [15]) and the American Food and Drug Administration (FDA) recommend for obsessive–compulsive disorder (OCD) treatment and which is also used off-label for other psychiatric disorders like (therapy-resistant) depression or anxiety [16]. Clomipramine, a tricyclic antidepressant with a rather unspecific neurotransmitter profile compared to SSRIs, is mostly considered second-line therapy, not least because of a higher risk of adverse side effects [17]. Yet, the clinical effectiveness of clomipramine as well as SSRIs in depression [18] and OCD [19] is high. 

We presume that SBL was reduced by a high-dose serotonergic effect via clomipramine and later escitalopram. We assumed an obsessive–compulsive character of our patient’s SBL behavior. The role of serotonin in the neurobiology of SI is subject of current research: lower levels of central–nervous serotonergic receptor binding in the prefrontal cortex [20] and reduced serotonin metabolites in the cerebrospinal fluid [21] have been demonstrated in patients showing SI behavior. We hypothesize that this could also be the case in SBL patients. Gene polymorphism of the serotonin transporter gene (5-HTTLPR) seem to mediate the psychopathology of borderline personality disorders in adult females but were unrelated to suicidal or SI behavior [22,23]. Despite these findings, the effectiveness of serotonergic medication regarding SI remains uncertain [24]. Pharmacogenetic polymorphisms, particularly regarding Cytochrome P450 metabolism, might have influenced the receptor binding profiles and thus the effectiveness of the serotonergic agents in our case as well. Previous literature describes the relevance of altered hepatic drug metabolism in psychopharmacology [25]. Pharmacogenetic polymorphisms were not assessed in our case; yet, they might explain the limited response to our treatment. However, pharmacogenetic assessments are not a main part of clinical procedures in psychiatry and are more commonly used in experimental and research contexts [26].

The increased risk of suicidal behavior following antidepressant medication compared to placebo is discussed in the literature. SSRIs, in particular, are postulated to increase the risk of suicidality among children, adolescents, and young adults [27], which is also hypothesized for clomipramine [28]. Although results are heterogenous in this regard [29], German professional information and the FDA highlight these adverse effects [30] and demand caution during dosing. This also includes escitalopram, which was used in our case. When re-dosing escitalopram after our patient’s rejection of clomipramine, we discussed borderline on-label (in Germany, up to 20 mg/d) vs. high-dose off-label escitalopram therapy for an even stronger serotonergic effect. Yet, we chose high-dose escitalopram on the edge of on-label treatment with 20 mg/d; higher off-label treatment was avoided in order to maintain drug safety and avoid adverse effects such as an increase in suicidality or SBL behavior during the initial treatment phase. 

Our patient showed recurring deliberate manipulative and concealing behavior, stealing and ordering sharp objects for SBL. Therefore, an intriguing question arose regarding the tolerance of diagnostic blood sampling in this context. There was uncertainty about whether she would avoid blood diagnostics because of fear or inner tension being ‘triggered’. On the other hand, the hospital team hesitated to fuel her ambitions of getting in touch with blood collection instruments and blood itself, thereby running the increased risk of repeated SBL. Our patient had also reported media content with intravenous drug use as a trigger of her SBL urge. Yet, blood samplings were necessary for control diagnostics under medication and monitoring of her iron-deficiency anemia; they were conducted under high precautionary measures in order to prevent her from stealing and concealing material for later SBL. Our patient herself did not report suffering from diagnostic blood sampling, yet her manipulativeness had to be considered.

This report has several limitations: as we can only report one clinical case of LFS and SBL, the evidence is not high. Further, we accompanied our patient through multiple inpatient and outpatient treatment stages, which allowed us a detailed evaluation of symptom changes during her weight increase. We were not able to include pharmacogenetic examinations into our clinical procedure, not least because of the limited benefit–cost ratio. The patient additionally received psychotherapeutic support (yet, without DBT-specific content) during her inpatient treatment. This and other psychiatric medications (e.g., daridorexant) limits the focus on specific pharmacologic agents.

Specific psychotherapeutic interventions seem to be the most effective treatment strategy for self-injury behavior. As postulated in the literature, SBL seems to be more than an expression of self-harm but a specific psychopathological entity with high treatment resistance. Yet, SBL- and LFS-affected patients could profit from a pharmacological-targeting approach. Our case report provides evidence that high-dose serotonergic (potentially off-label) medication with clomipramine and/or escitalopram poses a beneficial treatment approach and should be considered as a pharmacologic option for patients with SBL-related self-harm and suicidal intentions. Although SBL is a rare psychopathology, physicians and therapists involved in psychiatric diagnostics and treatment should be sensitised to the possible positive effects of high-dose serotonergic antidepressants among young SBL-affected patients. 

## Figures and Tables

**Table 1:** Therapeutic drug monitoring of clomipramine.

	Initial Dosing Stage	Dosage Increase	Steady State
Dosage dispended	0-0-150 mg/d	150-0-150 mg/d	150-0-150 mg/d
Drug Serum Level [µg/L] of…			
Clomipramine	72	148	106
Desmethyl–Clomipramine	131	353	307
Sum Score Clomipramine + Desmethyl-Clomipramine	203	501	413

Notes: Reference for all metabolites and sum score: 230–450 µg/L, Desmethyl–Clomipramine: Active steady-state metabolite of clomipramine.

## Data Availability

The datasets generated during and/or analyzed during the current study are available from the corresponding author on reasonable request.
